# Common Risk Factors Add to Inherited Thrombophilia to Predict Venous Thromboembolism Risk in Families

**DOI:** 10.1055/s-0039-1677807

**Published:** 2019-01-28

**Authors:** Pierre Suchon, Noemie Resseguier, Manal Ibrahim, Alexia Robin, Geoffroy Venton, Marie-Christine Barthet, Dominique Brunet, Noemie Saut, Marie-Christine Alessi, David A. Trégouët, Pierre E. Morange

**Affiliations:** 1 Laboratory of Haematology, La Timone Hospital, Marseille, France; 2 C2VN, Aix Marseille University, Marseille, France; 3 Support Unit for Clinical Research and Economic Evaluation, Assistance Publique - Hôpitaux de Marseille, Marseille, France; 4 EA3279 Self-perceived Health Assessment Research Unit, Aix-Marseille University, Marseille, France; 5 Aix-Marseille Université, TAGC Campus de Luminy, Marseille, France; 6 Department of Hematology and Cellular Therapy, AP-HM, Conception Hospital, Marseille, France; 7 Bordeaux Population Health Research Center, University of Bordeaux, Bordeaux, France

**Keywords:** family medical history, single nucleotide polymorphism, risk assessment, thrombophilia, venous thromboembolism

## Abstract

The clinical venous thromboembolism (VTE) pattern often shows wide heterogeneity within relatives of a VTE-affected family, although they carry the same thrombophilia defect. It is then mandatory to develop additional tools for assessing VTE risk in families with thrombophilia. This study aims to assess whether common environmental and genetic risk factors for VTE contribute to explain this heterogeneity. A total of 2,214 relatives from 651 families with known inherited thrombophilia were recruited at the referral center for thrombophilia in Marseilles, France, from 1986 to 2013. A thrombophilia screening was systematically performed in all included relatives. According to the severity of the thrombophilia defect, individuals were split into three groups: no familial defect, mild thrombophilia, and severe thrombophilia. In addition, common genetic factors (ABO blood group and 11 polymorphisms selected on the basis of their association with VTE in the general population) were genotyped. Furthermore, body mass index and smoking were collected. VTE incidence was 1.74, 3.64, and 6.40 per 1,000 person-years in individuals with no familial defect, mild thrombophilia, and severe thrombophilia, respectively. Five common risk factors were associated with VTE in this population: obesity, smoking, ABO blood group, and *F11* _rs2036914 and *FGG* _rs2066865 polymorphisms. These common factors were then included into a three-level risk score. The score was highly efficient for assessing VTE risk in mild thrombophilia patients by identifying two groups with different VTE risk; individuals with low score had the same risk as individuals with no familial defect whereas individuals with high score had the same risk as individuals with severe thrombophilia. An overall score including the five items plus the thrombophilia status was built and displayed an area under the receiver operating characteristic curve of 0.702 for discriminating VTE and non-VTE relatives. In conclusion, integrating common environmental and genetic risk factors improved VTE risk assessment in relatives from families with thrombophilia.

## Introduction

Venous thromboembolism (VTE), including deep vein thrombosis (DVT) and pulmonary embolism (PE), is the third cause of cardiovascular death. 1 The genetic part plays an important role as the heritability of VTE has been assessed to be 50 to 60%. 2 3 4 In clinical practice, when an inherited disorder is suspected a thrombophilia testing consisting of the search for the five following defects is performed: antithrombin (AT), protein C (PC) and protein S (PS) deficiencies, factor V Leiden (FVL), and the G20210A prothrombin mutation (PTM). 5 When a defect is diagnosed in a patient with a personal history of VTE, testing of family members is often performed to aid decision-making regarding future VTE prophylaxis in at-risk situations. However, usefulness of thrombophilia testing in assessing individual risk in families with inherited thrombophilia is largely debated as it does not properly assess the individual risk within a family. 6 7 Indeed, the test results do not clearly dichotomize carriers and noncarriers in terms of risk for a first episode of VTE. As a consequence, relatives harboring the family defect are usually considered at high risk, no matter the severity of the thrombophilia. Thus, the prevention strategy is mostly based on the result of the thrombophilia testing probably leading to an overprevention in particular in mild thrombophilia carriers (i.e., heterozygosity for FVL or PTM). On the contrary, relatives with a negative thrombophilia testing are often falsely considered at low risk.

The assessment of VTE risk in families with known inherited thrombophilia needs to be improved by acquiring more insight into the genetic and environmental risk factors. Other factors may then be taken into consideration when assessing individual risk. These could include both additional genetic risk factors much more common than those involved in the thrombophilia testing and environmental factors, the two main being obesity and smoking. 8 9 10 11 Assessment of obesity and smoking on the risk of VTE in families with known thrombophilia has never been performed yet.

The aim of the present study is to evaluate the influence of smoking, obesity, and newly identified common genetic factors on VTE risk in families with known thrombophilia with the ultimate goal of improving VTE risk assessment in family members.

## Methods

### Patients

Investigated families were part of the MARseilles FAmily Study on venous Thrombosis (MARFAST). The original MARFAST cohort has been extensively described previously. 12 Briefly, families were recruited between 1986 and 2008 at the referral center for thrombophilia in Marseilles, France. A family was included in the MARFAST cohort if it comprised at least two members with the following characteristics: one individual with a personal history of confirmed VTE and a positive thrombophilia testing (the proband) and a relative harboring the same defect (regardless his/her VTE history). All relatives referred to the center for thrombophilia were then included. Relatives were classified as first-degree relatives if they were a biological child or parent, or full sibling. Otherwise, they were considered as second-degree relatives. VTE was defined as a DVT and/or a PE. Superficial vein thrombosis was not included. A VTE episode was confirmed if objectively diagnosed by medical imaging: compression ultrasound, venography, ventilation/perfusion lung scan, spiral computed tomography or pulmonary angiography; or if the patient received full-dose anticoagulation for at least 3 months. A thrombophilia testing was systematically performed in all included relatives. Relatives were classified into three classes according to the severity of the thrombophilia: no familial defect, mild thrombophilia (FVL heterozygosity or PTM heterozygosity), and severe thrombophilia (AT, PC, PS deficiencies, FVL homozygosity, PTM homozygosity, and combined defects). The present study is an extension of the MARFAST cohort. The inclusions, using the same criteria, have been extended until 2013 at the same center. This MARFAST update included a total of 2,214 relatives (including 1,492 first-degree relatives) from 651 families, representing 151 more families and 940 more relatives than the previous analysis.

### Variables

A standardized questionnaire allowed the collection of clinical data during the consultation by a trained physician. The age, localization, and the triggering circumstances of VTE episodes were collected. A VTE episode was considered provoked if associated with an exposure within 3 months to exogenous risk factors: surgery, immobilization defined as bed confinement for at least 7 days, trauma, pregnancy, puerperium, combined oral contraceptive use, or malignancy. Otherwise, the episode was defined as unprovoked. The body mass index (BMI) was calculated during the consultation. Relatives were classified into three classes of BMI: <30 kg/m ^2^ , 30 to 35 kg/m ^2^ , and >35 kg/m ^2^ , based on the WHO classification of obesity. The smoking status, defined as current smoking (≥one cigarette per day) or nonsmoking, was determined either at the time of the episode for VTE relatives or at the time of the consultation for relatives with no personal history of VTE. Former smokers were classified as nonsmokers.

The characteristics of the probands were collected: age at the time of VTE and triggering circumstances at the time of first VTE. A thrombophilia testing was systematically performed in all relatives: AT, PC, and PS levels were measured as previously described and FVL and PTM were genotyped using light cycler technology (Roche Diagnostics, Indianapolis, Indiana, United States). 12 ABO blood group was genotyped and classified into three categories according to the level of risk previously estimated: A/B, AB, and O. The previous MARFAST investigation mainly relied on well-established risk factors for VTE and measurable coagulation-related biomarkers. Only three single nucleotide polymorphisms (SNPs) had been previously investigated in MARFAST, the *F11* polymorphisms (rs2036914 and rs2289252) and the *FGG8* _rs2066865. We here investigated the impact of eight additional SNPs whose association with VTE was supported by recent genome-wide association studies (GWASs): *F2* _rs3136516, *F5* _rs4524, *KNG1* _710446, *PROCR* _rs867186 and rs2069951, *SERPINC1* _rs2227589, *SLC44A2* _rs2288904, and *TSPAN15* _rs78707713.

### Statistical Analysis

Probands were excluded for the statistical analysis. First, a descriptive analysis was performed. Quantitative variables were described using means and standard deviations, and categorical variables were described using numbers and percentages. Comparisons of major clinical characteristics between VTE+ and VTE– groups were performed using chi-square test or Fisher's test when appropriate for categorical variables, and using Student's *t* -test or Mann–Whitney test when appropriate for quantitative variables. The incidence rate of VTE was estimated by dividing the number of VTE by the sum of the observation time for all participants. The observation time was defined as the period from birth until the first episode of VTE for participants who presented a VTE, or until the age at the visit for those who did not present a VTE episode. The incidence rate was expressed as number per 1,000 person-years (p-y). Univariate time-to-event analysis was first performed to assess the association between the risk of VTE over time and clinical and biological characteristics. A Cox regression model was used, including a frailty term to handle the family structure of the data (family identifier as a random effect).

Multivariate analyses were then performed to simultaneously integrate all characteristics that were associated with the risk of VTE with a *p* -value < 0.15 according to the univariate analyses. A multivariate frailty Cox model was built using a backward selection procedure to retain significant variables ( *p* < 0.05) and estimate adjusted hazard ratios (aHRs) with their 95% confidence interval (CI). The proportional hazards assumption was assessed by testing covariate interactions with quadratic function of time and checked graphically using Schoenfeld-type residuals. According to the value of the adjusted parameters (log HRs), a score of VTE risk was established by attributing 0, 1, or 2 points for each modality of the characteristics included in the final multivariate model. The incidence rate of VTE and its 95% CI were estimated according to this score to assess its validity. A receiver operating characteristic (ROC) curve was used to assess the global discriminative performances of the various explored scores, to distinguish VTE+ from VTE– patients. The area under the curve was estimated, with its 95% CI. Patients with missing data were excluded from the multivariate analysis.

All tests were two-sided. All *p* -values < 0.05 were considered significant. All analyses were performed with the R software.

All participants gave written informed consent, and the study met all institutional ethics requirement. The procedures employed were reviewed by the *Assistance Publique des Hopitaux de Marseille* institutional review committee.

## Results

A total of 2,214 relatives from 651 families were included. More than 60% of relatives had a positive thrombophilia testing, of whom 43.8 and 17.2% harbored a mild and a severe thrombophilia, respectively ( Supplementary Table S1 ). As expected, FVL was the most prevalent thrombophilia defect with a prevalence of 30.8% in relatives.

Main characteristics of the relatives according to VTE status are shown in Table 1 . In total, 246 (11.1%) presented with a VTE history. Among them, 113 had had an unprovoked episode (45.9%). The mean age at the end of the follow-up was 41.7 and 31.9 years ( *p* < 0.001) for VTE and VTE-free relatives, respectively.

**Table 1:** Main characteristics of the relatives according to the VTE status

Variable	VTE+ ( *n* = 246)	VTE– ( *n* = 1,968)	*p*
Female sex (%)	153 (62.2)	1,189 (60.4)	0.59
Mean follow-up, in years (SD)	41.7 (16.8)	31.9 (17.3)	< 0.0001
Thrombophilia screening			
No defect (%)	58 (23.6)	807 (41.0)	< 0.0001
Mild thrombophilia a (%)	113 (45.6)	856 (43.5)	
Severe thrombophilia b (%)	75 (30.5)	305 (15.5)	
ABO blood group			
A or B (%)	154 (66.4)	1,134 (60.0)	< 0.0001
AB (%)	25 (10.8)	99 (5.2)	
O (%)	53 (22.8)	657 (34.8)	
BMI < 30 kg/m ^2^ (%)	178 (79.1)	1,621 (93.0)	< 0.0001
BMI = 30–35 kg/m ^2^ (%)	36 (16.0)	91 (5.2)	
BMI > 35 kg/m ^2^ (%)	11 (4.9)	31 (1.8)	
Current smoking (%)	68 (27.6)	453 (23.1)	0.11
First VTE episode = PE (± DVT) (%)	53 (21.5)	–	–
Unprovoked VTE	113 (45.9)	–	–

a Mild thrombophilia: factor V Leiden heterozygosity or prothrombin mutation heterozygosity.

b Severe thrombophilia: antithrombin, protein C, protein S deficiencies, factor V Leiden homozygosity, prothrombin mutation homozygosity, and combined defects.

### Environmental Risk Factors

Grade 1 obesity defined as a BMI between 30 and 35 kg/m ^2^ and grade 2 obesity defined as a BMI above 35 kg/m ^2^ were associated with an increased risk of VTE. The incidence rate of VTE was 3.03 per 1,000 p-y for nonobese relatives (BMI < 30 kg/m ^2^ ), 6.38 per 1,000 p-y for relatives with grade 1 obesity, and 6.05 per 1,000 p-y for relatives with grade 2 obesity. As grade 1 and grade 2 incidences were comparable, the two categories were merged into one category of obese relatives (BMI > 30 kg/m ^2^ ) for further analyses. The overall incidence rate for obesity was thus 6.30 per 1,000 p-y (95% CI, 4.63–8.36).

The incidence rate was 3.19 per 1,000 p-y (95% CI, 2.74–3.69) and 4.00 per 1,000 p-y (95% CI, 3.11–5.07) for nonsmokers and smokers, respectively.

### Characteristics of First VTE in Probands

Neither the age nor the absence of triggering circumstances at the time of the VTE in probands was associated with VTE in relatives: 24.4% of VTE– relatives and 28.4% of VTE+ relatives had a proband with a history of VTE at a young age (<45 years) ( *p* = 0.19), and 60.5% of VTE– relatives and 60.6% of VTE+ relatives had a proband with a history of unprovoked VTE ( *p* = 0.94) (data not shown).

### Conventional Genetic Risk Factors

A thrombophilia was diagnosed in 76.1% of VTE+ relatives, whereas only 59% of VTE– relatives harbored a thrombophilia defect, the difference holding mainly in severe thrombophilia (30.5 vs 15.5%). The incidence rate of VTE increased with the severity of the thrombophilia defect: 1.74, 3.64, and 6.40 per 1,000 p-y in relatives with no defect, mild, and severe thrombophilia, respectively.

The incidence rate of VTE greatly varied according to the blood group, the highest incidence rate being observed for AB blood group relatives. Incidence rates were 2.29 per 1,000 p-y (95% CI, 1.71–2.99), 3.58 per 1,000 p-y (95% CI, 3.03–4.19), and 6.15 per 1,000 p-y (95% CI, 3.98–9.06) for O, A/B, and AB relatives, respectively.

### Genetic Polymorphisms Recently Identified as Risk Factors for VTE

Table 2 shows the results of the genotyping of the 11 selected polymorphisms. Three SNPs, *F11_* rs2036914 ( *p* = 0.003), *FGG* _rs2066865 ( *p* = 0.015), and *F11* _rs2289252 ( *p* = 0.036), were significantly associated with VTE risk.

**Table 2:** Genotyping of 11 selected SNPs reported to be associated with VTE

SNP	Risk allele	Genotype	VTE+	VTE–	*p* a
*FGG* rs2066865	T	CC	102	1,005	0.015
		CT-TT	112	777	
*F11* rs2036914	C	TT	37	470	0.003
		CT-CC	178	1,310	
*F11* rs2289252	T	CC	48	522	0.036
		CT-TT	162	1,230	
*F5* rs4524	A	GG	5	65	0.307
		GA-AA	214	1,729	
*SLC44A2* rs2288904	G	AA	12	103	0.882
		AG-GG	204	1,671	
*TSPAN15* rs78707713	T	CC	0	22	0.161
		CT-TT	224	1,799	
*PROCR* rs867186	G	AA	180	1,478	0.881
		AG-GG	38	321	
*F2* rs3136516	G	AA	86	628	0.211
		AG-GG	131	1,150	
*PROCR* rs2069951	A	GG	191	1,585	0.886
		GA-AA	26	209	
*SERPINC1* rs2227589	A	GG	154	1,364	0.491
		GA-AA	44	344	
*KNG1* rs710446	T	CC	41	357	0.702
		CT-TT	183	1,486	

Abbreviations: SNP, single nucleotide polymorphism; VTE, venous thromboembolism.

a *p* -Value calculated using a chi-square test.

A score combining all common genetic risk factors (11 polymorphisms + ABO blood group) was tested. Its predictive performance assessed according to the area under the receiver operating characteristics curve (AUC) was poor (AUC = 0.573; 95% CI, 0.529–0.616).

### Multivariate Analysis

The multivariate analysis comprised the thrombophilia status, obesity, smoking status, ABO blood group, *FGG* _rs2066865, *F11_* rs2036914, and *F11_* rs2289252, and a systematic adjustment for gender. The results are shown in Table 3 .

**Table 3:** Disease-free survival multivariate analysis with adjustment for age and gender and attributed score

Variables	*n* (%)	Hazard ratio (95% CI)	*p* -Value	Attributed score
No familial defect	647 (37.4)	1	–	–
Mild thrombophilia	794 (45.9)	1.91 (1.30–2.80)	< 0.0001	–
Severe thrombophilia	290 (16.8)	3.78 (2.50–5.73)	< 0.0001	–
BMI < 30 kg/m ^2^	1,589 (91.8)	1	–	0
BMI > 30 kg/m ^2^	142 (8.2)	1.78 (1.24–2.55)	0.002	1
No smoking	1,306 (75.4)	1	–	0
Current smoking	425 (24.6)	2.08 (1.51–2.86)	< 0.0001	1
Blood group O	565 (32.6)	1	–	0
Blood group A or B	1,068 (61.7)	1.37 (0.98–1.93)	0.067	1
Blood group AB	98 (5.7)	2.51 (1.50–4.22)	0.005	2
*F11* rs2036914–TT	418 (24.1)	1		0
*F11* rs2036914–CT/CC	1,313 (75.9)	1.55 (1.06–2.26)	0.023	1
*FGG* rs2066865–CC	957 (55.3)	1	–	0
*FGG* rs2066865–CT/TT	774 (44.7)	1.61 (1.21–2.13)	0.001	1

In the multivariate model, the highest aHR was observed for severe thrombophilia (aHR = 3.78 [95% CI, 2.49–5.73]). Among common risk factors, blood group AB and current smoking were associated with the highest levels of risk (aHR = 2.51 [95% CI, 1.50–4.22] and 2.08 [95% CI, 1.51–2.86], respectively). Obesity was associated with a 1.78-fold increase of VTE (95% CI, 1.24–2.55).

Only two SNPs remained significant in the multivariate model, *FGG* _rs2066865 and *F11_* rs2036914. The presence of at least one at risk allele was associated with an increased risk of VTE: aHR= 1.55 (95% CI, 1.06–2.26) and 1.61 (95% CI, 1.21–2.13) for *F11* _rs2036914 ( *p* = 0.001) and *FGG* _rs2066865 ( *p* = 0.023), respectively.

Interactions between all genetic and environmental factors selected for the multivariate analysis were tested. None were significant at a 0.05 threshold.

A risk stratification was then performed taking into account the five common risk factors for VTE: *F11* _rs2036914, *FGG* _rs2066865, ABO blood group, obesity, and smoking status.

**Fig. 1:**
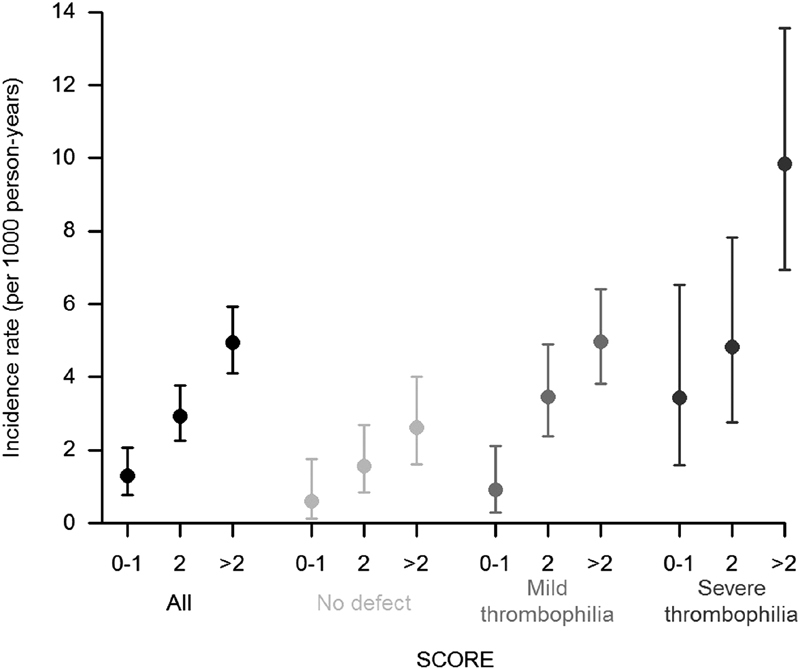
VTE incidence according to the score and the thrombophilia status. The score is calculated on the basis of five items: obesity according the WHO definition (BMI > 30 kg/m ^2^ ), smoking status, ABO blood group, *F11* rs2036914, and *FGG* rs2066865.

Observed HRs for the different components of the score being equally important, a simplified scoring method was applied: relatives were scored 0 or 1 point for each parameter but ABO. The scoring system was slightly different for ABO blood group because of the variety of ABO profiles and their associated HR: 0 for O blood group, 1 for A or B blood group, and 2 for AB blood group. After calculating the overall score, theoretically spanning from 0 to 6, relatives were classified into three groups: 0–1 point; 2 points; and 3 points or more. The VTE incidence rate was calculated in the three categories of risk in all relatives or according to the thrombophilia status ( Fig. 1 ; Supplementary Table S2 ).

A trend toward an increased incidence rate was observed according to the score: 1.30, 2.94, and 4.95 per 1,000 p-y in low-, medium-, and high-risk relatives, respectively ( *p* < 0.0001) ( Fig. 1 ; Supplementary Table S2 ). This observation held in all thrombophilia categories ( *p* = 0.04, < 0.0001, and 0.002 for no defect, mild, and severe thrombophilia, respectively). The combination of the information on this risk score together with the result of the thrombophilia testing led to an annual incidence rate ranging from 0.60 per 1,000 p-y for the combination of no defect and low risk score (score = 0 or 1) to 9.85 per 1,000 p-y for the combination of severe thrombophilia and high risk score (score ≥3) ( Fig. 1 ; Supplementary Table S2 ). It is noteworthy that the observed incidences in the mild thrombophilia group were quite heterogeneous in terms of risk. On the one hand, relatives with combination of mild thrombophilia/low risk score had a 0.91 per 1,000 p-y incidence, which was comparable to the combination of no familial defect/low risk score (0.60 per 1,000 p-y). On the other hand, relatives with combinations of mild thrombophilia/high risk score and severe thrombophilia/medium risk score displayed very similar incidences (4.98 and 4.83 per 1,000 p-y, respectively).

We then performed an overall time-to-event analysis taking into account both score and thrombophilia status, indicating that the observed effects were independent. Medium and high risk scores were associated with aHR of 2.31 (1.35–3.97) and 4.23 (2.54–7.04), respectively (global *p* -value < 0.0001), as compared with low risk score. Similarly, mild and severe thrombophilia were associated with aHR of 2.02 (1.38–2.95) and 3.88 (2.58–5.87), respectively (global *p* -value < 0.0001), as compared with no defect.

**Fig. 2:**
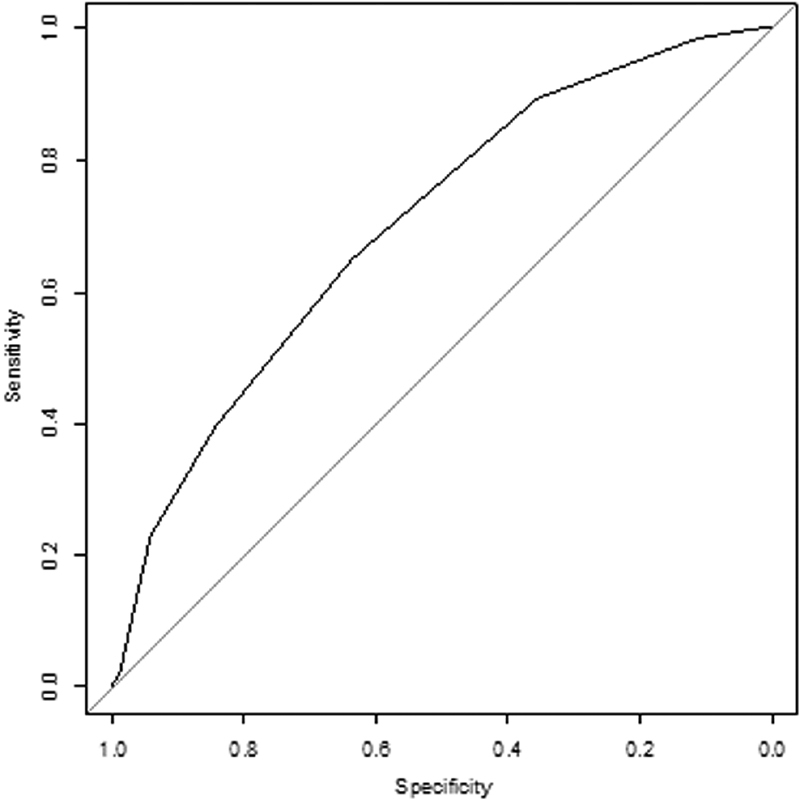
ROC curve of the risk score combining five common factors and the thrombophilia status. AUC = 0.702 (95% confidence interval, 0.666–0.739).

An overall predictive score including both common risk factors and the thrombophilia status was built. The ranking of common risk factors was the same as previously mentioned. One point was attributed for mild thrombophilia and 3 points for severe thrombophilia, according to the observed aHR in the multivariate analysis. The 6-item score demonstrated a good performance with an AUC of 0.702 (95% CI, 0.666–0.739). The ROC curve is shown in Fig. 2 . A subgroup analysis was then performed according to the triggering circumstances of the VTE episode. Similar results were obtained in both provoked and unprovoked episodes with respective AUC of 0.705 (95% CI, 0.656–0.754) and 0.728 (95% CI, 0.679–0.777) ( Supplementary Figs. S1 and S2 ).

The analysis was then performed according to the degree of kinship. Similar effects were observed when focusing the analysis in first-degree relatives only (data not shown).

## Discussion

Five common genetic and environmental factors were associated with VTE in families with thrombophilia: *F11* _rs2036914, *FGG* _rs2066865, ABO blood group, obesity, and smoking status. VTE risk assessment in families with heritable thrombophilia remains a major issue as relatives with no known defect harbor a two- to threefold increased risk of VTE compared with the general population and the vast majority of patients with a mild thrombophilia will not ever undergo a VTE episode. 13 14 15 This suggests that considering the familial defect testing only for assessing the individual risk in relatives is probably coarse. The assessment of VTE risk in families with inherited thrombophilia needs to be improved. This could be done by measuring the impact of common environmental and genetic risk factors. Although VTE is a complex disease, its environmental part has not been assessed in families with heritable thrombophilia. Among environmental risk factors, obesity and smoking are highly prevalent. Obesity is a well-established risk factor for VTE, whereas the relation between smoking and VTE has been debated for many years. 8 9 However, during the past 5 years, two large meta-analyses have been performed in the general population and reached the same conclusion that smoking is associated with VTE. 10 11 Compared with never smokers, the relative risk for current smokers was found to be about 1.2 both in the Cheng et al and Mahmoodi et al studies, respectively. 10 11

In the present study, we demonstrated that both common environmental and genetic factors modified the risk of VTE in families with thrombophilia. To the best of our knowledge, our study reports for the first time the incidence rate associated with obesity and smoking in families with thrombophilia. Obesity and smoking appeared to be significant predictors for VTE with associated HR of approximately 2. We here confirmed the association between smoking and increased VTE risk, with a slightly higher risk (∼2.1) observed in thrombophilia families. Very interestingly, the risk associated with these two environmental and thus modifiable factors was comparable to the risk associated with the presence of mild thrombophilia (HR, ∼1.9).

The characteristics of the probands' first VTE did not modulate VTE risk in relatives. These results differ somewhat with previous publication in which thrombosis at young age and unprovoked VTE in probands predict VTE in relatives. 16 However, only FVL and PTM were analyzed in this study and the vast majority of probands had no familial defect.

Among common genetic risk factors, we confirmed the previous results observed in the MARFAST study. 17 In the present extended MARFAST study, ABO blood group, *F11_* rs2036914, and *FGG* _rs2066865 were associated with HR ranging from 1.5 for *F11_* rs2036914 to 2.5 for blood group AB. Interestingly, *F11* _rs2036914 and *FGG* _rs2066865 are located in genes related to the coagulation cascade. They were previously reported to associate with plasma levels of the corresponding factors: the factor XI and the fibrinogen, respectively. 18 19 Of note, the latter is actually associated with a subunit of fibrinogen, the fibrinogen gamma, whose low levels were further demonstrated to associate with venous thrombosis. This opens the question of the clinical interest of measuring factor XI and fibrinogen gamma in families with thrombophilia.

To improve the assessment of VTE risk in families with inherited thrombophilia, we here proposed an effective additional tool based on the five common risk factors associated with VTE within the MARFAST families: smoking, obesity, ABO blood group, *F11_* rs2036914, and *FGG_* rs2066865. These five factors were then combined into a predictive score, which allowed the stratification of the risk in relatives, and then classified in low, medium, and high risk score patients. When considering the thrombophilia status only, the incidence rate was 1.74 per 1,000 p-y, 3.64 per 1,000 p-y and 6.40 per 1,000 p-y for no defect, mild thrombophilia and severe thrombophilia groups, respectively, whereas it actually ranged from 0.60 to 9.85 per 1,000 p-y when combining the thrombophilia testing and the risk score. Thus, the highest incidence observed in relatives with severe thrombophilia was not high enough for indicating primary prevention for VTE (VTE risk < bleeding risk under anticoagulant). As a consequence, such a score could not be used for identifying individuals that should benefit from primary prevention. On the contrary, the score is promising for identifying low-risk individuals. The assertion mainly holds in mild thrombophilia relatives. Even if most of them will remain disease-free, mild thrombophilia carriers are often considered at increased risk of VTE, in the absence of efficient tool. In this group of individuals, the global incidence rate was 3.64 per 1,000 p-y, whereas it actually ranged from 0.91 to 4.98 per 1,000 p-y according to the stratification. Very interestingly, the stratification identified 21% of the relatives harboring a low risk and an incidence rate comparable to the general population (0.91 vs. 1 per 1,000 p-y). 20 21 Similarly, 23% of no-defect relatives had low risk score and displayed a 0.60 per 1,000 p-y incidence. Altogether, the score could discriminate relatives with no defect or mild thrombophilia with “standard” VTE risk. The identification of those relatives could have positive impacts. For instance, combined oral contraceptives are contraindicated in mild thrombophilia carriers in most recommendations, whereas for some authors combined oral contraceptives could be used in the absence of additional risk factors. 22 It would be interesting to test this score in this specific population as individuals with low risk score might benefit from combined oral contraceptives use. Moreover, the score identified a group of relatives with no familial defect displaying a higher risk than the general population (incidence = 2.62 per 1,000 p-y). This former group of individuals might benefit from VTE prevention in at-risk situations. Of note, even if relatives with severe thrombophilia displayed a great amplitude of risk (incidence rate ranging from 3.44 to 9.85 per 1,000 p-y) according to the score, this stratification could not discriminate a group of relatives at low risk. As a consequence, those relatives with severe thrombophilia should probably be considered as a homogenous group of patients with a high risk of VTE according to most recommendations and thus benefit from prevention in at-risk situations. We further showed that the proposed score based on common risk factors was independent of the result of the thrombophilia testing, suggesting that it adds some valuable information for assessing the risk in relatives from families with known thrombophilia.

The performance of the overall score combining five common risk factors and the thrombophilia status was assessed. The AUC was 0.702 (95% CI, 0.666–0.739). Such predictive scores for VTE have been built in the general population. 23 24 25 However, only the score published by de Haan et al could be tested in our study according to the available data. It is a five-SNP risk score including three common SNPs (rs2036914 for ABO blood group, *FGG* _rs2066865, *F11* _rs2036914), FVL, and PTM. The score displayed 0.69 and 0.67 AUC in the MEGA study and the Leiden Thrombophilia Study, respectively. 23 In our cohort, the AUC of the de Haan score was 0.634 (95% CI, 0.594–0.675), which was slightly less than our proposed score that additionally integrates two common environmental risk factors (obesity and smoking) and severe thrombophilia.

To the best of our knowledge, the present study is one of the largest family studies on inherited thrombophilia published so far. However, this study shows some limits. In first instance, missing data led to a loss of power. Indeed, only 197 relatives with VTE could be tested out of the 242 included because of missing data, predominant on BMI (21 relatives). This lack of power could explain the absence of association between SNPs recently identified in GWASs and VTE in the families. In addition, we conducted a single-center study, which limits the generalizability of the results. An external validation study is mandatory. Another limitation of the study is the difference between VTE-free and VTE+ patients in terms of duration of follow-up (respectively, 32 and 42 years). However, the chosen analysis required to hypothesize that the censorship was not informative (time-to-event analysis based on likelihood). In addition, a subgroup analysis was performed in relatives under 45 years of follow-up displaying similar results as the whole cohort (data not shown). Finally, the diagnosis of AT, PC, and PS deficiencies was based on plasma assays, not on DNA sequencing. Nevertheless, the genetic nature of the deficiency can be assumed from the presence of at least two affected members (including the propositus) in the included families.

In conclusion, our results showed the impact of smoking and obesity on the risk of VTE in families with inherited thrombophilia. Taking into account common risk factors (both environmental and genetics) for VTE in families with inherited thrombophilia could be very helpful for assessing individual risk, per se or in addition to the thrombophilia testing. Independent and medico-economic studies are now mandatory to validate and define the indications of the score.

## Footnotes

*
*Equally contributed to the article.*
