# Supplementary material for: Common Risk Factors Add to Inherited Thrombophilia to Predict Venous Thromboembolism Risk in Families
Source: TH Open. 2019 Jan 28;3(1):e28–35. doi: 10.1055/s-0039-1677807 (PMC6524901; doi:10.1055/s-0039-1677807)
Supplement: Supplementary file 1 — Supplementary Material [file 10-1055-s-0039-1677807-s180058.pdf]

Supplementary Table S1 Results of the thrombophilia screening in relatives

Thrombophilia defect	Relatives, *n* (%)
FVL[a]	681 (30.8)
PTG20210A[b]	320 (14.5)
Antithrombin deficiency	30 (1.4)
Protein C deficiency	157 (7.1)
Protein S deficiency	79 (3.6)
Combined defects	82 (3.7)
No defect	865 (39.1)
Mild thrombophilia[c]	969 (43.8)
Severe thrombophilia[d]	380 (17.2)

[a]Factor V Leiden.
[b]G20210A prothrombin mutation.
[c]Factor V Leiden heterozygous or G20210A prothrombin mutation heterozygous.
[d]Antithrombin, protein C, protein S deficiencies, factor V Leiden homozygous, G20210A prothrombin mutation homozygous, combined defects.

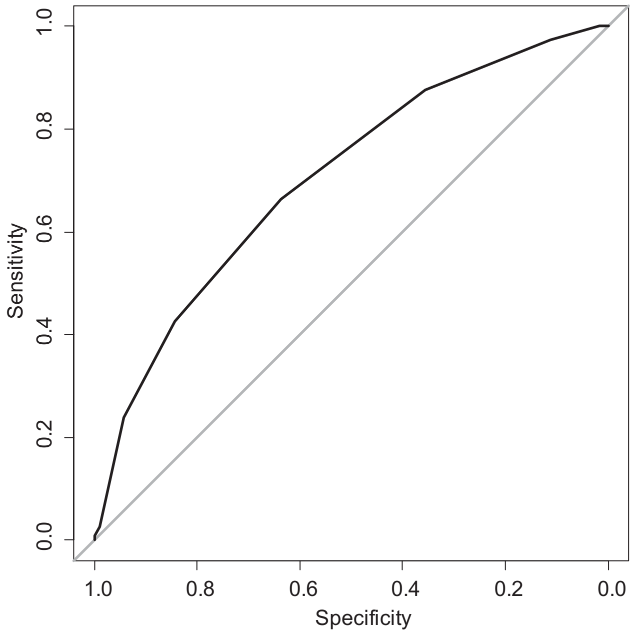

Fig. S1 ROC curve of the risk score combining five common factors and the thrombophilia status (provoked episodes). AUC = 0.705 (95% confidence interval, 0.656–0.754).

Supplementary Table S2 Venous thromboembolism incidence in relatives according to the score and the thrombophilia status

Population	Number of events	Follow-up (y)	Incidence[a] (95% CI)	HR (95% CI)	*p*-Value
All	197	57,918	3.40		
Score = 0–1 (*n* = 387)	17	13,116	1.30 (0.76–2.07)	1	<0.0001
Score = 2 (*n* = 629)	61	20,783	2.94 (2.25–3.77)	2.23 (1.30–3.85)	
Score ≥ 3 (*n* = 715)	119	24,019	4.95 (4.11–5.93)	4.09 (2.44–6.84)	
No defect	37	21,291	1.74	–	–
Score = 0–1 (*n* = 150)	3	5,010	0.60 (0.12–1.75)	1	0.0202
Score = 2 (*n* = 252)	13	8,255	1.57 (0.84–2.69)	2.57 (0.73–9.05)	
Score ≥ 3 (*n* = 245)	21	8,026	2.62 (1.62–4.00)	4.26 (1.26–14.27)	
Mild thrombophilia	98	26,944	3.64	–	–
Score = 0–1 (*n* = 164)	5	5,492	0.91(0.30–2.12)	1	<0.0001
Score = 2 (*n* = 276)	32	9,214	3.47 (2.38–4.90)	4.11 (1.55–10.89)	
Score ≥ 3 (*n* = 354)	61	12,238	4.98 (3.81–6.40)	7.23 (2.81–18.61)	
Severe thrombophilia	62	9,683	6.40	–	–
Score = 0–1 (*n* = 73)	9	2,614	3.44 (1.58–6.53)	1	
Score = 2 (*n* = 101)	16	3,314	4.83 (2.76–7.83)	1.24 (0.54–2.86)	0.0007
Score ≥ 3 (*n* = 116)	37	3,755	9.85 (6.95–13.56)	3.01 (1.45–6.26)	

Abbreviations: 95% CI, 95% confidence interval; HR, hazard ratio.
[a]Per 1,000 person-years.

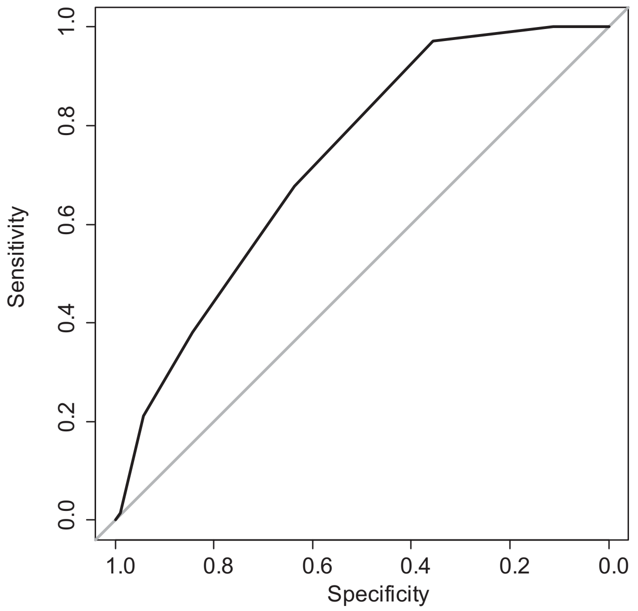

Fig. S2 ROC curve of the risk score combining five common factors and the thrombophilia status (unprovoked episodes). AUC = 0.728 (95% confidence interval, 0.679–0.777).
